# Dual Mechanisms of the Diazepine-Benzimidazole Derivative, DAB-19, in Modulating Glutamatergic Neurotransmission

**DOI:** 10.3390/ijms26115299

**Published:** 2025-05-30

**Authors:** Maxim V. Nikolaev, Irina M. Fedorova, Oxana V. Chistyakova, Tatiana Yu. Postnikova, Kira Kh. Kim, Mikhail Yu. Dron, Aleksey V. Zaitsev, Denis B. Tikhonov

**Affiliations:** Sechenov Institute of Evolutionary Physiology and Biochemistry, Russian Academy of Sciences, 194223 Saint Petersburg, Russia; fmedfstud@gmail.com (M.V.N.); fediri44@mail.ru (I.M.F.); chiosana@yandex.ru (O.V.C.); tapost2@mail.ru (T.Y.P.); kirakim2000@gmail.com (K.K.K.); neuro.mike@yahoo.com (M.Y.D.); aleksey_zaitsev@mail.ru (A.V.Z.)

**Keywords:** synaptic transmission, transmitter release, sodium channels, pharmacological modulation

## Abstract

The search for novel compounds with anticonvulsant properties remains a key focus in neuropharmacology. Recently, the diazepine-benzimidazole derivative, DAB-19, has emerged as a promising candidate due to its demonstrated anxiolytic and analgesic effects. In this study, we investigate the mechanisms underlying DAB-19’s activity, focusing on its impact on glutamatergic transmission, a key target in the pathophysiology of various central nervous system disorders. Intriguingly, while DAB-19 suppressed evoked glutamatergic transmission in rat brain slices, it simultaneously enhanced spontaneous neurotransmission. Further experiments on glutamatergic neuromuscular synapses in fly larvae revealed two distinct mechanisms: calcium-dependent potentiation of glutamate release and inhibition of spike propagation via blockade of voltage-gated sodium channels. The latter effect was directly confirmed in rat brain neurons. Given its action on sodium channels, we tested DAB-19 in the pentylenetetrazole model, where it delayed seizure onset but did not prevent seizures. These findings position DAB-19 as a multifaceted compound with significant therapeutic potential.

## 1. Introduction

Pharmacological correction of CNS disorders, such as anxiety, chronic pain, epilepsy, seizures, and depression, is crucial for modern medicine, which offers a wide range of drugs for the prevention and treatment of these conditions [1,2,3,4]. Effective drugs demonstrate a wide variety of molecular targets. Antidepressants are widely prescribed to treat major depressive disorder, anxiety disorders, chronic pain, and certain addictions. In clinical practice, serotonin reuptake inhibitors, monoamine oxidase inhibitors (MAOIs), and benzodiazepine derivatives are commonly used, but these drugs often cause adverse side effects, primarily CNS inhibition, such as daytime sleepiness, muscle weakness, emotional blunting, headaches, dizziness, and ataxia. Anticonvulsants target voltage-gated sodium and calcium channels, GABA_A_ receptors, synaptic vesicle proteins, and glutamate receptors [5].

Glutamatergic transmission, which plays a key role in the excitatory processes in the CNS, is one of the principal targets in various CNS disorders [6,7,8]. The NMDA receptor antagonist ketamine [9], initially developed as a dissociative anesthetic, is also used as an antidepressant [10]. Ketamine acts as a rapid-onset antidepressant, though its effects are transient [11]. The anticonvulsant perampanel acts as a noncompetitive AMPA receptor antagonist [12]. Efficacy and clinical tolerance of perampanel is related to its specific allosteric mechanism of action [13]. Anticonvulsant activity of sodium channel blockers is due to the high activity-dependence of their action; they weakly block the channels in the case of infrequent activations, but readily inhibit frequently activated channels [14,15,16]. Thus, the molecular mechanism of drug action is essential for their effects in physiological and pathological conditions. In addition, many drugs exert complex effects through multitarget mechanisms [5]. The pharmacological profile of tricyclic antidepressants [17,18] and fluoxetine [19] includes inhibition of ionotropic glutamate receptor activity. The anticonvulsant phenytoin also inhibits AMPA-type glutamate receptors [20], though the contribution of this mechanism to its anticonvulsant activity remains unclear. All these examples underscore the need for careful analysis of the pharmacological profiles and mechanisms of action of novel promising drugs.

**Figure 1:**
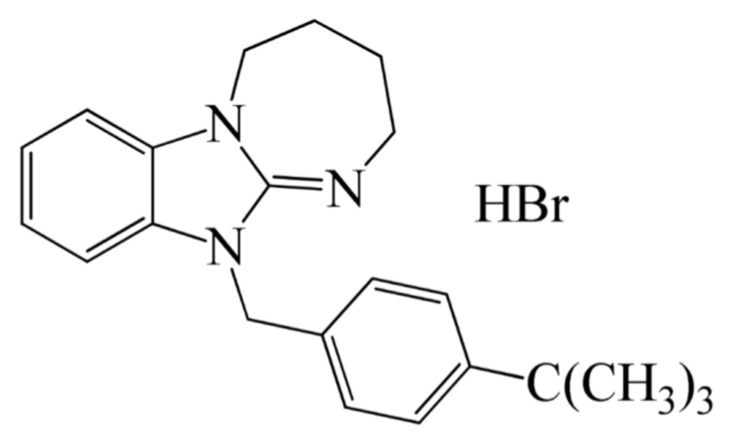
Chemical structure of 1-(4-tert-butylbenzyl)-2,3,4,5-tetrahydro[1,3]diazepino[1,2-a]benzimidazole hydrobromide (DAB-19).

The diazepino[1,2-a]-benzimidazole derivatives, which combine pharmacologically privileged benzimidazole and diazepine scaffolds, are promising as anxiolytic and analgesic agents [21,22]. Recently described compound DAB-19 (Figure 1) demonstrated anxiolytic properties in the elevated plus maze and open field tests, while analgesic effects were evaluated using the tail-flick and hot-plate methods. Additionally, DAB-19’s influence on motor function was examined in grid, wire, and rotarod tests. DAB-19 exhibited prominent analgesic effects. It also enhanced exploratory behavior, demonstrated mood-stabilizing properties, and reduced aggression and anxiety-like behavior [23].

While the effects of DAB-19 appear promising, its molecular targets remain unknown. In the present study, the effects of DAB-19 on glutamatergic transmission were analyzed using electrophysiological approaches. Experiments were performed on rat brain slices and neurons, as these objects are routinely used in analogous studies. Patch clam and extracellular recordings allowed complex characterization of the effects. We also studied DAB-19 action on glutamatergic transmission in fly larvae neuromuscular junction. Use of this preparation has allowed us to confirm the results obtained on rat brains and to reveal interesting peculiarizes of action, which were not clearly seen in rat brains. Taken together, our results demonstrate a dual effect of the compound: calcium-dependent stimulation of synaptic release, and inhibition of voltage-gated sodium channels. In glutamatergic synapses, DAB-19 inhibits evoked transmission while potentiating spontaneous release. Given its action on sodium channels, we hypothesized that DAB-19 could exhibit anticonvulsant activity, which was subsequently tested in the pentylenetetrazole model. This unique pharmacological profile positions DAB-19 as a promising candidate for further investigation.

## 2. Results

### 2.1. DAB-19 Inhibits Field Responses in the Rat Hippocampus

**Figure 2:**
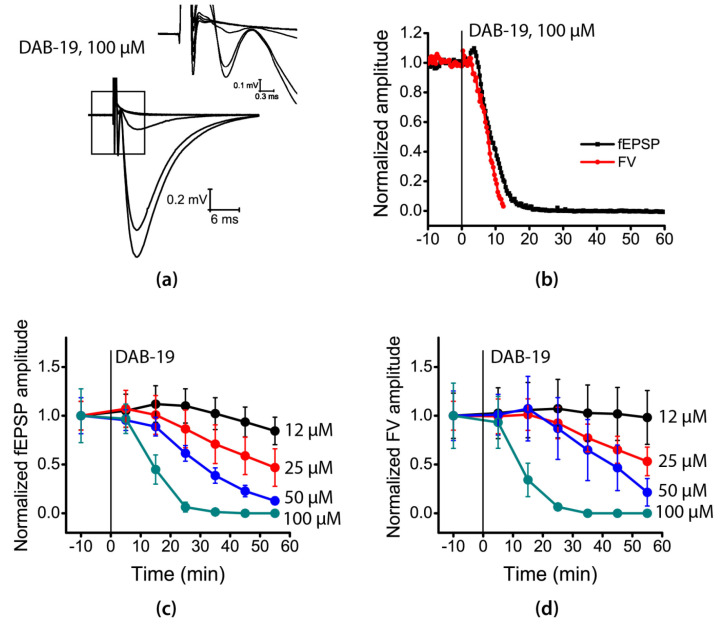
Effects of DAB-19 on field potentials in the rat hippocampus. (**a**) Representative traces of field potentials before (black) and after application of 100 µM DAB-19 (colored lines), recorded every 10 min. Inset shows fiber volley (FV) at higher temporal resolution. (**b**) Time course of FV amplitude changes following application of different DAB-19 concentrations (mean ± SEM, normalized to control). (**c**) Time course of field excitatory postsynaptic potential (fEPSP) changes (mean ± SEM, normalized to control). (**d**) Synchronous reduction in FV and fEPSP amplitudes following 100 µM DAB-19 application.

To identify the molecular targets and mechanisms of action of DAB-19, we began with classical experiments on glutamatergic transmission in the hippocampus. DAB-19 application concentration dependently reduced both fiber volley (FV) and field excitatory postsynaptic potential (fEPSP) amplitudes (Figure 2a). At 12 µM, DAB-19 had no significant effect on FV or fEPSP amplitudes (Figure 2b,c), whereas a marked reduction in both FV and fEPSP amplitudes occurred synchronously at 50–100 µM (Figure 2d). These data suggest that DAB-19 suppresses synaptic transmission by impairing presynaptic action potential generation.

To identify the site of DAB-19’s action, we investigated short-term synaptic plasticity, which strongly depends on neurotransmitter release probability [24]. We applied paired-pulse stimulation with a 50 ms interstimulus interval. The paired-pulse ratio (PPR) was calculated as the ratio of the second to first fEPSP amplitudes. Analysis revealed no significant differences in PPR compared to baseline values (repeated measures ANOVA: F(6, 39) = 0.83; *p* = 0.55).

### 2.2. Opposite Effects of DAB-19 on Evoked and Spontaneous Transmission in Rat Cortex

**Figure 3:**
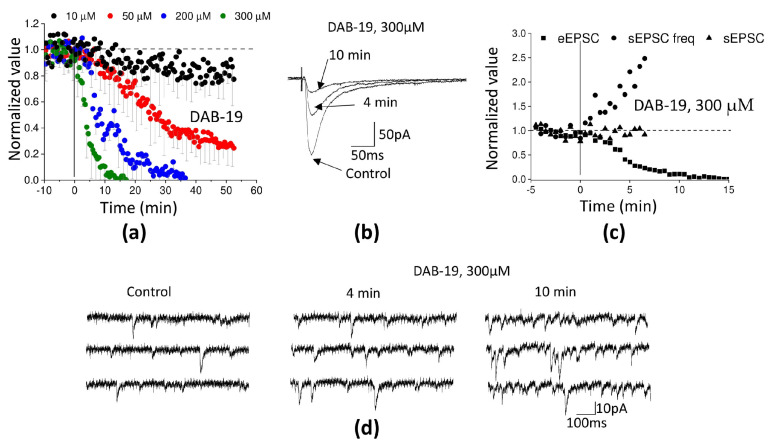
Action of DAB-19 on evoked and spontaneous glutamatergic synaptic transmission in rat cortical neurons. (**a**) Time course of eEPSC amplitude changes at different DAB-19 concentrations (mean ± SEM, normalized to baseline). (**b**) Representative averaged eEPSC traces (7 consecutive recordings) in control and after 4/10 min of 300 µM DAB-19 application. (**c**) Time course of eEPSC amplitude, sEPSC amplitude, and frequency in a representative experiment. (**d**) Original sEPSC recordings in control and after 4/10 min of 300 µM DAB-19 application.

We performed detailed analysis of DAB-19’s effects on synaptic transmission in cortical slices using the patch-clamp technique. Figure 2a shows the time course of a representative experiment in L2/3 pyramidal cells. At 10 µM, DAB-19 had no noticeable effect; the amplitude of evoked post-synaptic current (eEPSC) decreased by only 11 ± 5% (*n* = 6) over 50 min of recording. This decrease was comparable to response rundown in control experiments (10 ± 5%, *n* = 8). At 50 µM, DAB-19 induced slow, incomplete eEPSC inhibition (67 ± 3% reduction over 50 min, *n* = 5). At 200–300 µM, inhibition was nearly complete and developed faster (Figure 3a,b). The eEPSC amplitude decreased to 10% of control within 19 ± 6 min (*n* = 5) at 200 µM and 9 ± 2 min (*n* = 5) at 300 µM, respectively.

To reveal the site of action, we studied the effect of 300 µM DAB-19 on spontaneous release (Figure 3c,d). We did not detect changes in the amplitude of spontaneous post-synaptic current (sEPSC) (*n* = 5; *p* < 0.05, paired *t*-test). This finding suggests that the postsynaptic effect of DAB-19 is absent or weak. Surprisingly, DAB-19 caused a prominent increase in sEPSC frequency, which increased 2–3 times during 10 min of application. The opposite effects on spontaneous and evoked release are very unusual. It is known that α-latrotoxin causes [25] massive spontaneous release, which is accompanied by a decrease in evoked release due to the depletion of synaptic vesicles [26]. However, α-latrotoxin causes a 20–30-fold increase in eEPSC frequency and about a 3-fold decrease in eEPSC amplitude [27]. In contrast, the moderate effect of DAB-19 on sEPSC frequency in our experiments was accompanied by almost complete inhibition of eEPSC. It is unlikely that such an increase in spontaneous release causes strong depletion of synaptic vesicles. An alternative possibility is that DAB-19 exerts independent effects on spontaneous and evoked release.

### 2.3. Effects of DAB-19 on Neuromuscular Glutamatergic Transmission in Fly Larvae

To resolve the issue of the unusual opposite effects of DAB-19 on evoked and spontaneous release, we studied its action on glutamatergic neuromuscular transmission in fly larvae *Calliphora vicina*. In control experiments, the amplitude and frequency of spontaneous EPSCs were 0.24 ± 0.17 nA (*n* = 20) and 6.8 ± 3.5 Hz (*n* = 20), respectively.

**Figure 4:**
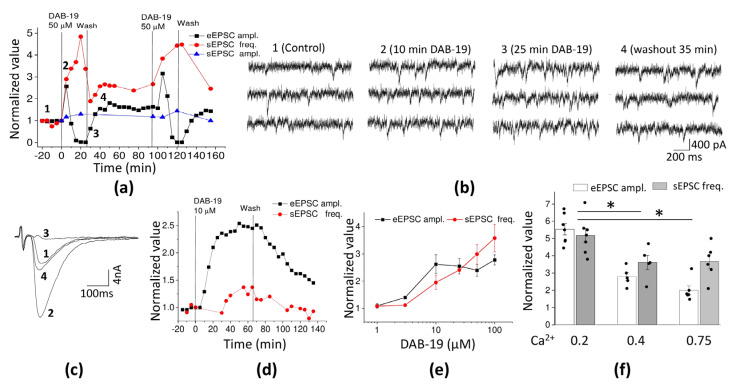
Effects of DAB-19 on glutamatergic neuromuscular transmission in fly larvae. (**a**) Time course of eEPSC amplitude, sEPSC amplitude, and frequency changes (normalized to control) during 50 µM DAB-19 application and washout. (**b**) Representative sEPSC recordings in control and during DAB-19 application. (**c**) Representative eEPSC recordings showing transient potentiation followed by inhibition. Labels 1-4 mark the stages of experiment in panel (**a**). (**d**) Effects of 10 µM DAB-19 on eEPSC amplitude and sEPSC frequency. (**e**) Concentration-dependence of DAB-19 effects on sEPSC frequency and maximal eEPSC amplitude. (**f**) Calcium-dependence of 50 µM DAB-19 effects on eEPSC amplitude and sEPSC frequency at different extracellular Ca^2+^ concentrations. Asterisks mark significant differences (*p* < 0.05).

The time course of a typical experiment is shown in Figure 4a. Representative recordings of eEPSCs and sEPSCs are given in Figure 4b,c. After the control recording, DAB-19 (50 µM) was added to the extracellular solution. As in the case of rat neurons, the amplitude of sEPSCs remained unaffected (*n* = 6; *p* < 0.05, paired *t*-test), while the frequency of sEPSCs increased. The effect reached a maximum value of 3.62 ± 1.04 (*n* = 6) relative to the control. Surprisingly, the eEPSC amplitude increased sharply after DAB-19 application and then dropped to almost zero values. The average values for initial transient potentiation and maximal inhibition (relative to control) were 2.8 ± 0.6 (*n* = 6) and 0.016 ± 0.005 (*n* = 6), respectively. The effects on eEPSC amplitude and sEPSC frequency were reversible, and the entire experiment could be repeated (see Figure 4a).

The results obtained from rat brain synapses and fly neuromuscular synapses are generally similar. In both preparations, DAB-19 caused a prominent increase in sEPSC frequency. In both cases, the sEPSC amplitude remained unchanged, while the eEPSCs were strongly inhibited. However, eEPSC inhibition in rat brain neurons developed monotonically, whereas in fly neuromuscular transmission the eEPSC inhibition was preceded by a transient rise in amplitude. This biphasic behavior of eEPSCs suggests the presence of two independent mechanisms. The initial amplitude increase is likely mediated by the same mechanism that causes the increase in sEPSC frequency. The subsequent inhibition of eEPSCs appears to involve a distinct mechanism that does not affect spontaneous release. The transient increase in eEPSC amplitude is observable because the inhibitory mechanism develops more slowly.

If the complex action of DAB-19 is due to distinct mechanisms, they can also be discriminated by their concentration dependencies. In contrast to rat brain slices, 10 µM DAB-19 caused an increase in sEPSC frequency (1.5 ± 0.3 relative to control, *n* = 8) and potentiated the eEPSC amplitude (2.1 ± 0.6, *n* = 8), but the inhibitory phase of the effect on eEPSC amplitude was absent (Figure 4d). The concentration dependence of DAB-19’s effects is shown in Figure 4e. At 1 µM, DAB-19 was ineffective, while 3 µM caused modest but significant effects. For concentrations ≥ 50 µM, the inhibitory phase became apparent, and the maximal amplitude during the initial potentiating phase was used to quantify the effect.

Regrettably, fitting the concentration dependencies proved impractical. We observed no clear saturation for sEPSC frequency. At high drug concentrations, resolving individual sEPSCs became problematic. For eEPSC amplitude, the developing inhibitory effect partially masked the potentiation. The kinetics of the effect were also concentration-dependent. Maximal amplitude potentiation with 10 µM DAB-19 was reached within 40 min, whereas the effects of 50 and 100 µM developed within 10 min or less. Inhibition of eEPSC amplitude developed over 40 min at 50 µM, while at 100 µM DAB-19, the effect developed twice as fast.

Comparison of the data obtained between the two preparations demonstrates qualitative similarity (both effects are present) but significant quantitative differences. The eEPSC inhibition is pronounced in both preparations at concentrations of 50 µM and above. The facilitation of release, responsible for increased sEPSC frequency, occurs at lower concentrations in fly neuromuscular transmission compared to rat brain transmission. As a result, we were able to analyze this effect in more detail.

Facilitation of release could result from enhanced calcium entry into nerve terminals. If this is the case, the effect should depend on extracellular calcium concentration. We tested the calcium dependence of DAB-19’s effects on sEPSC frequency and eEPSC amplitude using DAB-19 concentrations of 10 and 100 µM, with calcium concentrations reduced to 0.2 mM or increased to 0.75 mM. No calcium dependence was found for the effects of 10 µM DAB-19 on either sEPSC frequency or eEPSC amplitude (unpaired *t*-test, *n* = 5–7). However, the effects of 50 µM were calcium-dependent, being more pronounced at 0.2 mM calcium than at 0.4 and 0.75 mM (one-way ANOVA with Tukey’s test, *n* = 5–7, Figure 4f). Thus, our results suggest that DAB-19 may facilitate calcium entry into nerve terminals, stimulating both spontaneous and evoked transmitter release.

**Figure 5:**
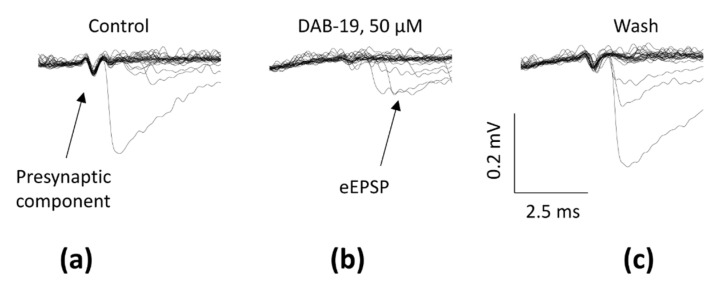
Extracellular recordings of neuromuscular transmission in fly larvae. Superimposed traces (*n* = 30) showing (**a**) control responses with clear presynaptic components; (**b**) complete elimination of presynaptic components during 100 µM DAB-19 application; (**c**) recovery after washout.

The mechanism producing eEPSC inhibition must be fundamentally different. The simplest explanation is that DAB-19 prevents spike propagation, possibly by blocking sodium channels. To test this hypothesis, we performed extracellular recordings to allow detection of presynaptic depolarization. As shown in Figure 5, presynaptic components were clearly visible under control conditions but virtually disappeared after the application of 50 µM DAB-19. The effect was reversible (see Figure 5c). Note that the results from extracellular recordings in fly neuromuscular junctions (Figure 5) and rat brain slices (Figure 2) are consistent. In both preparations, DAB-19 abolished presynaptic components, suggesting the same mechanism of evoked response inhibition.

### 2.4. Inhibition of Voltage-Gated Sodium Channels in Rat Brain Neurons

**Figure 6:**
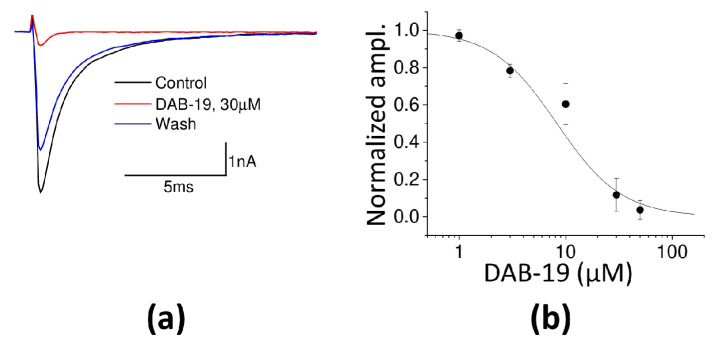
Inhibition of voltage-gated sodium channels by DAB-19 in isolated rat neurons. (**a**) Representative sodium current traces in control, during 30 µM DAB-19 application, and after washout. (**b**) Concentration–response curve for sodium current inhibition (mean ± SEM, *n* = 5). Solid line represents Hill equation fit (IC_50_ = 8.3 ± 1.4 µM).

The hypothesis that DAB-19 inhibits voltage-gated sodium channels was tested in whole-cell patch-clamp experiments on isolated hippocampal CA1 pyramidal neurons. In these experiments, potassium channels were blocked (10 mM tetraethylammonium chloride and 4 mM 4-aminopyridine) along with calcium channels (0.1 mM CdCl_2_). Sodium channels were activated by 30 ms long voltage steps from −110 mV to −10 mV. After control recordings (Figure 6a), DAB-19 was added to the bath solution. The full effect developed within approximately 2 min, and subsequent washout resulted in restoration of the response within 5 min. Finally, 0.5 μM of tetrodotoxin (TTX) was applied to eliminate currents mediated by voltage-gated sodium channels. Traces recorded with TTX were subtracted from those recorded without it. Figure 6a shows strong inhibition of sodium current by 30 µM DAB-19 (0.12 ± 0.08 relative to control, *n* = 5). The IC_50_ value, derived from fitting the concentration-inhibition data to the Hill equation, was 8.3 ± 1.4 μM (Figure 6b). Thus, direct action of DAB-19 on voltage-gated sodium channels was unambiguously demonstrated.

### 2.5. Suppression of Seizure Activity in Rats by DAB-19 in the Pentylenetetrazole (PTZ) Test

The multifaceted pharmacological profile of DAB-19, including its inhibition of voltage-gated sodium channels, prompted us to investigate its systemic anticonvulsant potential. We assessed the effects of two DAB-19 doses (2.9 mg/kg and 10 mg/kg) against PTZ-induced seizures in Wistar rats, with 300 mg/kg sodium valproate (VPA) as a reference anticonvulsant and saline as a vehicle control (*n* = 10 per group).

In control animals, PTZ administration elicited generalized seizures in 90% of rats (9/10), with one mortality. Both DAB-19-treated groups showed similar seizure incidence (8/10), while VPA provided robust protection, preventing generalized seizures in 90% of cases (1/10 with seizures). No mortality occurred in treatment groups.

**Figure 7:**
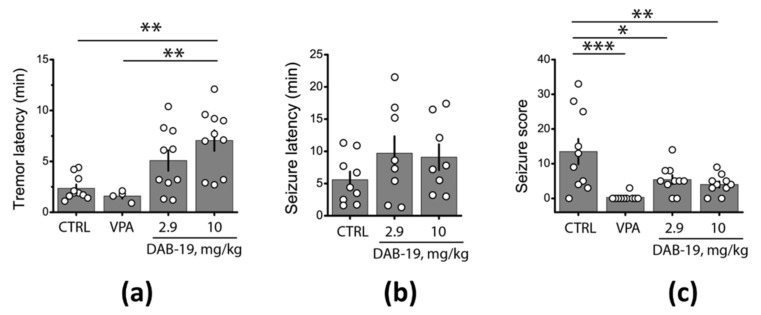
Anticonvulsant effects of DAB-19 in the PTZ test. (**a**) Latency to tremor onset (scores 1–2). (**b**) Latency to generalized seizures (scores ≥ 3). (**c**) Cumulative seizure severity scores. Data shown as mean ± SEM (columns) with individual data points (circles). * *p* < 0.05, ** *p* < 0.01, *** *p* < 0.001 vs. control (one-way ANOVA with Tukey’s test). Abbreviations: VPA (300 mg/kg valproate).

Significantly, DAB-19 prolonged the latency to tremor onset (scores 1–2; F(3,30) = 7.55, *p* < 0.001, Figure 7a), although no significant intergroup differences were observed in latency to generalized seizures (scores ≥ 3; F(2,22) = 1.31, *p* = 0.29, Figure 7b; VPA excluded due to low seizure incidence). Furthermore, DAB-19 reduced cumulative seizure severity scores compared to controls (13.5 ± 3.7 in controls vs. 5.4 ± 1.3 (2.9 mg/kg DAB-19) and 4.0 ± 0.9 (10 mg/kg)), while VPA almost completely suppressed seizure severity (0.3 ± 0.3; F(3,36) = 7.82, *p* < 0.001, Figure 7c). These findings demonstrate that DAB-19 exerts moderate anticonvulsant activity.

## 3. Discussion

In the present work, we studied the effects of the diazepine-benzimidazole derivative DAB-19 on glutamatergic neurotransmission. The multiple systemic effects of this compound revealed in previous studies [21,23] required detailed analysis to determine its mechanisms of action. Our results demonstrate that DAB-19 robustly suppresses neuronal excitability, as shown by its concentration-dependent reduction in FV and fEPSP amplitudes in the rat hippocampus. The synchronous decline in both FV and fEPSP amplitudes suggests that DAB-19 impairs action potential propagation in presynaptic axons, likely via blockade of voltage-gated sodium channels. This mechanism is consistent with observations for other sodium channel inhibitors, such as methylmercury, which similarly reduces afferent input by suppressing axon recruitment and FV amplitude [28].

To further investigate presynaptic contributions, we employed the PPR protocol. The lack of PPR modulation suggests that the inhibitory effect does not result from altered presynaptic calcium dynamics during evoked release, which typically mediate PPR changes [24]. The absence of PPR modulation by DAB-19 mirrors the effects of local anesthetics, which suppress synaptic transmission by selectively inhibiting axonal sodium channels [29]. Notably, DAB-19 shows differential effects compared to lamotrigine, which enhances PPR and exhibits use-dependent presynaptic inhibition [30]. These findings indicate that DAB-19 primarily attenuates axonal signaling without altering short-term plasticity. This distinct mechanism differentiates DAB-19 from classical anticonvulsants and suggests its potential as a therapeutic agent targeting axonal hyperexcitability.

Patch clamp recordings in rat brain slices revealed strong inhibition of eEPSC amplitudes without affecting sEPSC amplitudes, suggesting a presynaptic site of inhibition. Similar results were observed at glutamatergic neuromuscular junctions in fly larvae. Most importantly, the sodium channel blockade hypothesis was directly confirmed in experiments measuring sodium currents in isolated rat brain neurons, which were readily blocked by DAB-19. As expected for voltage-gated sodium channel blockers, anticonvulsant activity was confirmed through suppression of PTZ-induced seizure activity in rats.

However, some of our findings do not allow us to characterize DAB-19 as a simple sodium channel blocker. In both synaptic preparations examined, we detected a strong increase in sEPSC frequency following DAB-19 application. Moreover, at fly larval neuromuscular synapses, low DAB-19 concentrations caused parallel increases in both sEPSC frequency and eEPSC amplitude. Higher concentrations produced a transient increase in eEPSC amplitude that was followed by profound inhibition. This potentiating component was found to be calcium-dependent. These results strongly suggest that DAB-19 has a multitarget mechanism of action. While we cannot currently identify the specific target responsible for the potentiating effects, we note that the synaptic release machinery comprises numerous components, and potential mechanisms for enhancing spontaneous release are highly diverse.

The complex multitarget action that we have demonstrated for DAB-19 is not unique to this compound. Many low-molecular-weight drugs affect multiple targets, creating complex pharmacological profiles. In addition to the examples mentioned in the introduction, we recently showed that lamotrigine and phenytoin likely inhibit insect potassium channels, causing membrane depolarization that facilitates calcium entry into nerve terminals [31]. Similarly, certain serine protease inhibitors have been characterized as potent blockers of both NMDA and AMPA receptor channels [32,33], as well as acid-sensing channels [34].

Systemic effects of multitarget drugs are difficult to predict. We hypothesized that DAB-19 might have antiepileptic effects and tested this hypothesis using the PTZ-induced seizure model. Our results demonstrate that DAB-19 did not prevent seizures, although it delayed seizure onset and reduced cumulative severity scores. The dose-independent nature of DAB-19’s action likely reflects its multitarget pharmacology, combining sodium channel blockade with modulation of spontaneous release. This profile contrasts with that of lamotrigine (LTG), which primarily inhibits sodium channels but shows limited effects on PTZ-induced clonus latency [35].

Importantly, DAB-19 appears to offer unique advantages for addressing epilepsy-associated comorbidities. Preclinical behavioral tests demonstrated its anxiolytic and cognitive-enhancing properties, such as reduced anxiety-like behavior (evidenced by increased center entries in the open field test) and diminished aggression [23]. These effects differ markedly from the cognitive impairments associated with VPA [36]. Although LTG may partially alleviate epilepsy-related cognitive deficits [37], DAB-19’s dual modulation of excitability—suppressing pathological synchronization through sodium channel blockade while enhancing spontaneous glutamate release—may help preserve synaptic plasticity, a crucial mechanism underlying cognitive function.

This dual mechanism is consistent with therapeutic strategies targeting glutamatergic system hyperactivity. For example, downregulation of brain-specific serine/threonine-protein kinase 1 reduces PTZ-induced seizures by modulating AMPA receptors [38]. Similarly, DAB-19’s calcium-dependent enhancement of spontaneous release may counteract pathological hyperexcitability while maintaining physiological neurotransmission, potentially offering a more balanced therapeutic approach for refractory epilepsy with neuropsychiatric comorbidities.

While DAB-19 demonstrated delayed seizure onset in the PTZ model, further studies are needed to evaluate its potential as an adjunctive therapy given its multitarget profile, The compound’s differential effects on evoked versus spontaneous release represent a particularly interesting phenomenon. While spontaneous release was previously considered merely a byproduct of evoked release with limited functional significance, recent studies have established its crucial role in shaping neuronal morphology and modulating nascent synaptic connections [39]. Furthermore, spontaneous and evoked neurotransmission may represent distinct neuronal signaling pathways that operate in spatially segregated manners [40].

In conclusion, we characterized the action of DAB-19 on glutamatergic synaptic transmission and revealed a dual mechanism that includes (i) inhibition of presynaptic spike propagation via block of voltage-gated sodium channels and (ii) calcium-dependent stimulation of glutamate release. Due to this unique complex action, DAB-19’s system effects may exhibit unique characteristics warranting detailed investigation. Future studies should focus on optimizing dosing regimens and exploring potential synergies with existing medications, especially for patients with drug-resistant epilepsy and associated neuropsychiatric comorbidities.

## 4. Materials and Methods

### 4.1. Animals

Male Wistar rats were used throughout this study. For electrophysiological experiments, three-week-old rats were used, while mature male Wistar rats weighing 230–260 g were utilized for the pentylenetetrazole test. All animals were obtained from the Rappolovo Animal Facility (Russia) and housed under standard laboratory conditions at controlled room temperature with ad libitum access to food and water. All experimental procedures complied with the ARRIVE guidelines and EU Directive 2010/63/EU for the protection of animals used for scientific purposes. We implemented all possible measures to both minimize the number of animals used and alleviate potential suffering.

### 4.2. Slice Preparation and Electrophysiological Recordings

Electrophysiological experiments were performed on acute brain slices obtained from rats. Horizontal slices (350 µm thick) were prepared using an HM 650 V vibratome (Microm International, Walldorf, Germany) in ice-cold (0–4 °C) artificial cerebrospinal fluid (aCSF) containing (in mM): 126 NaCl, 24 NaHCO_3_, 2.5 KCl, 2 CaCl_2_, 1.25 NaH_2_PO_4_, 1 MgSO_4_, and 10 glucose (pH 7.4 when bubbled with carbogen). Slices were incubated in oxygenated (95% O_2_, 5% CO_2_) aCSF at 35 °C for 1 h before being transferred to a recording chamber maintained at 30 °C.

Local field potentials were recorded from the CA1 stratum radiatum using glass microelectrodes (0.2–1.0 MΩ resistance) positioned in the CA1 stratum radiatum. Signals were amplified using a Model 1800 amplifier (A-M Systems, Carlsborg, WA, USA) and digitized with an NI USB-6211 ADC/DAC converter (National Instruments, Austin, TX, USA). Data acquisition was performed using WinWCP v5.7.8 software (University of Strathclyde, Glasgow, UK).

For each slice, we measured the amplitudes of fEPSPs, which reflect summed postsynaptic responses in pyramidal neuron dendrites, and FV amplitudes, representing presynaptic action potentials. Stimulation intensity (100 µs duration pulses) was individually calibrated for each slice, ranging from 25–300 µA in 25 µA increments, to establish fEPSP amplitudes at 40–50% of the maximum response. Throughout experiments, this optimized stimulation intensity (typically 50–150 µA) remained constant.

Evoked excitatory postsynaptic currents were recorded from L2/3 pyramidal cells in the medial prefrontal cortex using an EPC-10 patch-clamp amplifier (HEKA Elektronik, Lambrecht, Germany). During recordings, slices were continuously perfused with oxygenated (95% O_2_, 5% CO_2_) aCSF maintained at room temperature (22–24 °C). Neurons were identified using an upright microscope (BX51WI, Olympus, Tokyo, Japan) equipped with a 40× water-immersion objective and differential interference contrast (DIC) optics. Series resistance was typically 15–20 MΩ and remained stable (<20% change) for cells included in analyses. Patch pipettes were fabricated from borosilicate glass (WPI, Sarasota, FL, USA) and filled with solution contained (in mM): 127 CsSO_3_CH_2_, 10 NaCl, 5 EGTA, 10 Hepes, 4 Mg-ATP, 0.3 Na-GTP, and 10 phosphocreatine (pH adjusted to 7.3 with CsOH, 295 mOsm L^−1^). Extracellular stimulation used bipolar pulses (100 µs duration, ≤100 μA; A360 stimulus isolator, WPI). Stimulating electrodes, filled with aCSF, were positioned 150 μm from the recorded soma. Only one neuron was recorded per slice. DAB-19 was bath-applied to aCSF.

Currents through native Na_v_ channels were studied on CA1 pyramidal cells using an EPC-10 patch-clamp amplifier (HEKA Elektronik, Lambrecht, Germany). The neurons were isolated by vibrodissociation method [41]. Membrane voltage was clamped at −110 mV and ion channels were activated with a short (30 ms) depolarizing step to −10 mV (stimulation frequency was 0.1 Hz). The extracellular solution contained (in mM) 143 NaCl, 5 KCl, 2.5 CaCl_2_, 10 D-glucose, 10 TEA-Cl, 4 4-AP, 0.1 CdCl_2_, 10 HEPES, pH 7.4 (adjusted with HCl), t = 22–24 °C. The patch pipettes were filled with solution containing (in mM) 100 CsF, 40 CsCl, 5 NaCl, 0.5 CaCl_2_, 5 EGTA, 10 HEPES, pH 7.2 (adjusted with CsOH). DAB-19 was added to the extracellular solution and applied to the cell body using the rapid perfusion system (RSC-200, BioLogic Science Instruments, Claix, France).

### 4.3. Pentylenetetrazole Test

Forty mature male Wistar rats (230–260 g) were randomly assigned to four experimental groups (*n* = 10 per group):

Control Group: Received a single intraperitoneal (i.p.) injection of PTZ (70 mg/kg) prepared in 0.9% saline to induce acute seizures. Injection volumes were individually calculated based on body weight.

Reference Group: Received sodium valproate (300 mg/kg in 0.9% saline, i.p.) 30 min prior to PTZ administration [42].

Experimental Groups: Received DAB-19 (2.9 or 10 mg/kg in pre-warmed 0.9% saline, i.p.) 30 min before PTZ challenge.

Seizure activity was evaluated by continuous video monitoring and scored using a modified Racine scale [43]: 0 indicates no behavioral changes; 1 refers to isolated/repetitive myoclonic jerks; 2 includes facial clonus (rhythmic mouth movements) progressing to neck clonus (head nodding/jerking); 3 involves forelimb clonus (uni/bilateral, synchronous/asynchronous) without rearing; 4 consists of forelimb clonus with rearing; 5 represents generalized clonic seizures (rearing, falling, jumping) progressing to tonic phases; and 6 indicates tonic extension.

Scores 0–2 represented minor/focal seizures; scores of ≥3 indicated generalized seizures. Behavioral assessments were conducted for 60 min post-PTZ injection.

Anticonvulsant activity was evaluated based on the ability of the compounds to prevent clonic and tonic components of seizures. The following parameters were recorded: (1) latency to minor/focal seizures (scores 1–2) and (2) latency to generalized seizures (scores of ≥3); (3) severity of generalized seizures (scores 3–6), expressed as a cumulative score: seizure score × duration (rounded up to the nearest whole minute) [44].

### 4.4. Fly Larvae Neuromuscular Preparation

Late third-instar larvae of *Calliphora vicina* (Diptera: Calliphoridae) were used for experiments. After dissection, the internal organs were removed, leaving only the muscles attached to the cuticle. Recordings were made from ventral longitudinal fibers. The ventral ganglion was excised, and the segmental nerves were stimulated via a suction electrode with pairs of suprathreshold pulses (1 ms duration). The stimulation frequency was 0.5 Hz to ensure stable responses.

The preparation was perfused with a saline solution containing (in mM): 172 NaCl, 2.5 KCl, 0.4 CaCl_2_, 5 MgCl_2_, 5 HEPES, and 52 sucrose. The pH was adjusted to 7.4 with NaOH or HCl. Experiments were performed at room temperature (21–23 °C). Under these conditions, the membrane potential ranged from −45 to −65 mV.

Intracellular eEPSCs and sEPSCs were recorded using the standard two-electrode voltage-clamp technique with pipettes filled with 2.5 M KCl (tip diameter: 1–1.5 µm; resistance: 5–10 MΩ). The membrane potential was held at −60 mV. Postsynaptic currents were recorded using an Axoclamp 2B amplifier (Axon Instruments, Foster City, CA, USA). Data were filtered at 2 kHz and stored digitally. To minimize inter-signal variability, 10 sequential eEPSCs were averaged before analysis. Spontaneous EPSCs were collected between eEPSCs, and 100 sequential signals were averaged for amplitude analysis. Signals were aligned to the midpoint of their rising phase to reduce fluctuations.

### 4.5. Data Analysis and Statistics

Results are expressed as mean ± SEM from ≥5 independent experiments. Data were analyzed using Origin 9.1 (OriginLab, Northampton, MA, USA) after verifying normality distributions with Shapiro–Wilk tests. Within-group comparisons used paired two-tailed Student’s *t*-tests (drug vs. control). Between-group analyses employed independent samples *t*-tests or one-way ANOVA with Tukey’s post hoc correction for multiple comparisons (F(df1, df2) reported where applicable). Differences were considered significant at *p* < 0.05.

## Data Availability

The data presented in this study are available upon request from the corresponding author.
